# Differences in response to antiretroviral therapy in HIV-positive patients being treated for tuberculosis in Eastern Europe, Western Europe and Latin America

**DOI:** 10.1186/s12879-018-3077-x

**Published:** 2018-04-23

**Authors:** Yanink Caro-Vega, Anna Schultze, Anne Marie W. Efsen, Frank A. Post, Alexander Panteleev, Aliaksandr Skrahin, Jose M. Miro, Enrico Girardi, Daria N. Podlekareva, Jens D. Lundgren, Juan Sierra-Madero, Javier Toibaro, Jaime Andrade-Villanueva, Simona Tetradov, Jan Fehr, Joan Caylà, Marcelo H. Losso, Robert F. Miller, Amanda Mocroft, Ole Kirk, Brenda Crabtree-Ramírez

**Affiliations:** 1 Department of Infectious Diseases, Instituto Nacional de Ciencias Médicas y Nutrición, Salvador Zubirán, Vasco de Quiroga 15, Col. Belisario Domínguez sección XVI, Tlalpan, CP 14080 Mexico City, Mexico; 2 Department of Infection and Population Health, University College London Medical School, London, UK; 3 Centre for Health and Infectious Disease Research (CHIP), Department of Infectious Diseases, Finsencentret, Rigshospitalet, University of Copenhagen, Copenhagen, Denmark; 4 Department of Sexual Health, Caldecot Centre, King’s College Hospital, London, UK; 5 Department of HIV/TB, TB hospital 2, St. Petersburg, Russia; 6 Clinical Department, Republican Research and Practical Centre for Pulmonology and TB, Minsk, Belarus; 7 Infectious Diseases Service, Hospital Clinic – IDIBAPS. University of Barcelona, Barcelona, Spain; 8 Department of Infectious Diseases INMI “L. Spallanzani”, Ospedale L Spallanzani, Rome, Italy; 9 HIV Unit, Hospital J.M. Ramos Mejia and CICAL, Fundación IBIS, Buenos Aires, Argentina; 10 Infectious Diseases Service, Hospital Civil de Guadalajara, Guadalajara, Mexico; 11 Dr Victor Babes’ Hospital of Tropical and Infectious Diseases, Bucharest AND ‘Carol Davila’ University of Medicine and Pharmacy, Bucharest, Romania; 12 Division of Infectious Diseases and Hospital Epidemiology, University Hospital Zurich, Zurich, Switzerland; 13 Agencia de Salud Pública de Barcelona: Programa Integrado de Investigación en Tuberculosis de SEPAR (PII-TB); Centro de Investigación Biomédica en Red de Epidemiología y Salud Pública (CIBERESP), Barcelona, Spain; 14 Centre for Clinical Research in Infection and Sexual Health, Institute for Global Health, University College London, London, UK

**Keywords:** HIV, Tuberculosis, ART, Efavirenz, Protease inhibitor, Outcomes, Eastern Europe

## Abstract

**Background:**

Efavirenz-based antiretroviral therapy (ART) regimens are preferred for treatment of adult HIV-positive patients co-infected with tuberculosis (HIV/TB). Few studies have compared outcomes among HIV/TB patients treated with efavirenz or non-efavirenz containing regimens.

**Methods:**

HIV-positive patients aged ≥16 years with a diagnosis of tuberculosis recruited to the TB:HIV study between Jan 1, 2011, and Dec 31, 2013 in 19 countries in Eastern Europe (EE), Western Europe (WE), and Latin America (LA) who received ART concomitantly with TB treatment were included. Patients either received efavirenz-containing ART starting between 15 days prior to, during, or within 90 days after starting tuberculosis treatment, (efavirenz group), or other ART regimens (non-efavirenz group). Patients who started ART more than 90 days after initiation of TB treatment, or who experienced ART interruption of more than 15 days during TB treatment were excluded. We describe rates and factors associated with death, virological suppression, and loss to follow up at 12 months using univariate, multivariate Cox, and marginal structural models to compare the two groups of patients.

**Results:**

Of 965 patients (647 receiving efavirenz-containing ART, and 318 a non-efavirenz regimen) 50% were from EE, 28% from WE, and 22% from LA. Among those not receiving efavirenz-containing ART, regimens mainly contained a ritonavir-boosted protease inhibitor (57%), or raltegravir (22%). At 12 months 1.4% of patients in WE had died, compared to 20% in EE: rates of virological suppression ranged from 21% in EE to 61% in WE. After adjusting for potential confounders, rates of death (adjusted Hazard Ratio; aHR, 95%CI: 1.13, 0.72–1.78), virological suppression (aHR, 95%CI: 0.97, 0.76–1.22), and loss to follow up (aHR, 95%CI: 1.17, 0.81–1.67), were similar in patients treated with efavirenz and non-efavirenz containing ART regimens.

**Conclusion:**

In this large, prospective cohort, the response to ART varied significantly across geographical regions, whereas the ART regimen (efavirenz or non-efavirenz containing) did not impact on the proportion of patients who were virologically-suppressed, lost to follow up or dead at 12 months.

**Electronic supplementary material:**

The online version of this article (10.1186/s12879-018-3077-x) contains supplementary material, which is available to authorized users.

## Background

Globally, tuberculosis (TB) is the commonest opportunistic infection in HIV-infected patients, especially in resource-constrained settings [1]. Mortality in co-infected patients varies across regions as reported previously [2, 3], however, in most regions TB is the most common cause of death among the HIV-infected adult population [4–6]. Prospective cohort studies demonstrated that HIV patients co-infected with TB have an increased risk of death [5], and reduced mortality with appropriate TB treatment and initiation of antiretroviral therapy (ART) [7, 8]. Additionally, in patients with TB who present with advanced immunodeficiency (CD4 count < 200 cells/mm^3^), early initiation of ART (< 8 weeks after TB treatment initiation) increases survival, as demonstrated in several large clinical trials [9–12]. Efavirenz-based regimens were used in all of these studies [9–12]. The efficacy of other ART regimens in TB/HIV co-infected patients, and the relative efficacy of rifampicin- or rifabutin-containing regimens in patients receiving ART remains poorly studied [13–15]. Hence, National and regional guidelines provide minimal guidance on such ART regimens used concomitantly with tuberculosis treatment [1, 16–22].

On the other hand, late presentation of HIV-positive adult patients into care is common in both resource-constrained and high-income settings [23–25] and episodes of opportunistic infection including TB, may occur within the first few months after initiation of ART [8]. In most parts of the world with limited resources and a high burden of TB, efavirenz remains the preferred agent as the third component of ART regimens, as it has no clinically-significant interaction with rifampicin [1, 26]. However, tolerability and non-nucleoside reverse transcriptase inhibitor resistance may preclude use of efavirenz. In 2007, the WHO recommended increasing the ritonavir boosting dose to 400 mg every 12 h with lopinavir when concomitantly used in combination with rifampicin [21] however this has been associated with poor tolerability and hepatotoxicity [27–29]. More recently, raltegravir was reported to be an adequate alternative to efavirenz in TB/HIV co-infected patients in a phase 2 trial [30]. Therefore, CDC and WHO both indicate raltegravir to be the preferred option when efavirenz is contraindicated, or the use of rifabutin when ritonavir-boosted protease inhibitors are necessary [1, 16, 18, 31]. However, access to rifabutin and raltegravir in low- and middle-income countries, is often difficult or impossible [30]. The aim of this study was to assess clinical outcomes (mortality, virological suppression, loss to follow-up during the first year following TB diagnosis) across different regions (Eastern Europe, Western Europe, and Latin America) among HIV-positive adult patients treated with efavirenz and non-efavirenz containing ART regimens.

## Methods

### Study population

The TB:HIV study is a collaboration between TB and HIV clinicians from 19 countries in Europe and Latin America. Patients aged 16 years or older were included if they were HIV-positive and were diagnosed with TB between January 1, 2011, and December 31, 2013. Patients with confirmed TB (*Mycobacterium tuberculosis* on culture or PCR), probable TB (Acid-Fast Bacilli on ‘smear’ or granulomatous inflammation on biopsy specimens) and presumptive TB (empiric TB treatment initiated, and with a TB diagnosis not subsequently ruled out) were included in the study. Details of the TB:HIV study have been published elsewhere [2]. For the main analysis, those who initiated tuberculosis treatment and ART concomitantly were analyzed. Data on TB and HIV disease, including demographic, clinical, laboratory parameters, and clinical outcomes were collected prospectively on standardized case report forms (http://www.cphiv.dk). Due to the observational nature of the study, all decisions regarding use of tuberculosis and ART treatment regimens was at the discretion of individual clinicians.

### Statistical analyses

In this study, patients were categorized into two groups according to their ART regimen. In the efavirenz group patients who initiated an efavirenz-containing ART regimen within 3 months of starting TB therapy, or who were receiving it (at least in the 2 weeks before TB diagnosis) were included. Patients, who interrupted ART for more than 15 days while on anti-TB drugs, were excluded. In the non-efavirenz group, patients receiving ART but not containing efavirenz within the above time frame were included. Patients in this group included those in receipt of ritonavir-boosted protease inhibitors, triple nucleoside reverse transcriptase inhibitors, integrase inhibitors or nevirapine. Baseline was considered the date anti-TB treatment was commenced. Demographic and clinical characteristics of patients at baseline were described by receipt of ART in each region and for all study participants. Patients were recorded as dead, in care with virological suppression, in care without virological suppression, in care without viral load information, or lost to follow up at 12 months after their TB diagnosis. A viral load < 400 copies/mL between 8 and 12 months after baseline was considered as virological suppression. Patients with a last visit date reported before 12 months of follow-up were considered lost to follow up. All patients were included in analysis of time to outcome, and were censored 12 months after the TB diagnosis.

The main outcome was mortality during the first year following TB diagnosis comparing efavirenz and non-efavirenz containing ART regimens, using three different statistical methods to account for potential confounders and bias: inverse probability weighting, Cox models and marginal structural models. For the first method, Kaplan-Meier survival curves were estimated using inverse weighting. Weights were calculated from a logistic model to predict efavirenz use, adjusting by traditional confounders: region, age, gender, CD4 count at TB diagnosis, type of TB, route of HIV transmission, naïve status, rifamycin use (rifampicin or rifabutin), and multidrug-resistant TB (MDR-TB). In the second method, a Cox model was used to estimate risk of death using the same co-variables, stratifying by naïve status. Continuous variables such as age and CD4 cell count were included in the models using splines with three knots to fit a non-linear and less biased relationship with the outcome [32]. Finally, a marginal structural model was used to estimate the effect of both ART regimes on mortality in the presence of varying time co-variables (time-dependent confounders), as well as type of rifamycin used, CD4 count, and resistance to anti-TB drugs (variables that could affect choice of ART regimen). With this method, simulation of a clinical trial comparing two regimes of ART in HIV individuals co-infected with TB, controlling by time updated measurements of the confounders, was attempted. To achieve this, a monthly data set was generated for each patient selected from the time of TB diagnosis until 12 months of follow-up, recording all the socio-demographic and clinical endpoints month by month.

Following standard use of marginal structural models, patients who did not receive ART at initiation of TB treatment were included in this analysis since, as they were on tuberculosis treatment, they had the potential to be started by clinicians on ART with any regimen, as in local clinical practice [33]. The dataset was expanded in order to have the same patients and their characteristics in either of two possible regimens of ART (efavirenz-containing, or not), and artificial censoring was created to keep only the time in which patients did not leave the regimen. To adjust for a potential bias due to unbalance of co-variables in each regimen stabilized weights from two logistic models were built to predict use of ART controlling by time, age, gender, region, naive status, rifamycin use, injection drug use (IDU) status, type of TB (disseminated compared to pulmonary), CD4 count, and MDR-TB. The probability of death was estimated with a pooled logistic regression model using the ART regimen, time in months, region, CD4 count, gender, naïve status, MDR-TB status, with rifamycin receipt as a co-variable and with stabilized weights [33]. Sensitivity analysis was done including only ART-naïve patients. Cox models adjusted by traditional confounders were used, looking for factors related to virological suppression among patients at 12 months after diagnosis of TB who were in care with a viral load available, and who were lost to follow up.

## Results

Of 1389 patients in the study, 965 (69%) received ART during the predefined time-frame and were included in the main analysis: 647 were in the efavirenz group and 318 in the non-efavirenz group. We excluded 177 patients because they started ART after the time-frame (i.e. > 3 months after initiation of TB treatment), and 18 because of ART interruptions (> 15 days) during TB treatment. Baseline characteristics of participants stratified by ART regimen and ART status are shown in Table 1. Patients were treated with a rifamycin (rifampin or rifabutin) for a median of 7 months (IQR: 2–10). Patients had a median of one viral load measurement during the first year following ART initiation (IQR: 0–2).

**Table 1:** Demographic and clinical characteristics of patients at the time of starting TB therapy

	All (*N* = 965)		ART naïve (*N* = 518)		Non ART naïve (*N* = 447)	
	Efavirenz (*n* = 647)	non Efavirenz (*n* = 318)	Efavirenz (*n* = 420)	non Efavirenz (*n* = 98)	Efavirenz (*n* = 227)	non Efavirenz (*n* = 220)
Patient age at TB diagnosis (years)	37 (31–43)	37 (31–45)	37 (32–44)	35 (32–43)	36 (31–42)	38 (31–47)
Male^*^, n(%)	499 (77%)	206 (65%)	345 (82%)	73 (74%)	154 (68%)	133 (60%)
Ethnic group, n(%)^*+&^						
White	432 (68%)	182 (60%)	298 (73%)	61 (68%)	134 (60%)	121 (56%)
Hispanic	118 (19%)	43 (14%)	63 (15%)	10 (11%)	55 (25%)	33 (15%)
Black	47 (7%)	62 (20%)	26 (6%)	14 (16%)	21 (9%)	48 (22%)
Other	35 (6%)	18 (6%)	22 (5%)	5 (6%)	13 (6%)	13 (6%)
HIV risk, n(%)^*^						
non IDU	382 (59%)	211 (66%)	243 (58%)	56 (57%)	139 (61%)	155 (70%)
IDU	265 (41%)	107 (34%)	177 (42%)	42 (43%)	88 (39%)	65 (30%)
Region, n(%)^*+&^						
Eastern Europe	363 (56%)	118 (37%)	262 (62%)	50 (51%)	101 (44%)	68 (31%)
Western/Southern Europe	131 (20%)	144 (45%)	77 (18%)	39 (40%)	54 (24%)	105 (48%)
Latin America	153 (24%)	56(18%)	81 (19%)	9 (9%)	72 (32%)	47 (21%)
CD4^+^ count (cells/mm^3^)^*+^	96 (33–210)	118 (34–310)	82 (29–164)	60 (18–118)	140(48–297)	168(56–372)
Missing CD4 counts, n (%)	3 (< 1%)	1 (< 1%)	3 (1%)	1 (1%)	0(0%)	0(0%)
HIV RNA (copies/mL), n(%)^*^ < 400	76 (12%)	63(20%)	8 (2%)	2 (2%)	68 (30%)	61 (28%)
400–10 0000	39 (6%)	36 (11%)	18 (4%)	4 (4%)	21 (9%)	32 (14%)
> 10 0000	319 (49%)	121 (38%)	254(60%)	61 (62%)	65 (29%)	60 (27%)
Unknown	213 (33%)	98 (31%)	140 (33%)	31 (32%)	73 (32%)	67 (30%)
Haemoglobin (g/dL)	12 (10–14)	12 (10–14)	12 (10–14)	12 (10–13)	13 (10–14)	12 (10–14)
Disseminated TB^+^, n(%)	373 (58%)	189 (59%)	74 (76%)	237 (56%)	136 (60%)	115 (52%)
Resistance test performed	348 (54%)	169 (53%)	226 (54%)	49 (50%)	122 (54%)	120 (54%)
MDR-TB, n(%)°	68 (19%)	20 (12%)	47 (21%)	12 (24%)	21 (17%)	8 (7%)
Initial anti-TB drug, n(%)^*+&^						
Rifampicin	587 (91%)	208 (65%)	390 (93%)	70 (71%)	197 (87%)	138 (63%)
Rifabutin	13 (2%)	69 (22%)	9 (2%)	12 (12%)	4 (2%)	57 (26%)
No rifamicin	47 (7%)	41 (13%)	21 (5%)	16 (16%)	26 (11%)	25 (11%)
ART regimen, n (%)						
Efavirenz	647 (100%)	0 (0%)	420 (100%)	0 (0%)	227 (100%)	0 (0%)
Protease inhibitor	0 (0%)	181 (57%)	0 (0%)	62 (63%)	0 (0%)	119 (54%)
Integrase inhibitor	0 (0%)	70 (22%)	0 (0%)	23 (23%)	0 (0%)	47 (21%)
Other	0 (0%)	67 (21%)	0 (0%)	13 (13%)	0 (0%)	54 (25%)
Time between TB therapy and ART initiation (days)^&^	–	–	28 (17–49)	28 (15–34)	376 (47–1198)	1337 (199–3484)

*Note:* Continuous variables are reported as medians and interquartile ranges. ^*^p < 0.05 between patients receiving efavirenz and non-efavirenz -containing regimens of ART including the entire cohort. ^+^*p* < 0.05 between efavirenz and non-efavirenz-containing ART regimens, including only naïve patients

^&^*p* < 0.05 between efavirenz and non-efavirenz including only non-naïve patients. Time between TB diagnosis and ART initiation was estimated between non-naïve patients as TB diagnosis date less first ART date. °Percentage estimated relative to those with resistance test performed

*IDU* Injecting Drug User*,* MDR-TB Multi-drug resistant tuberculosis

### Clinical outcomes at 12 months

**Fig. 1:**
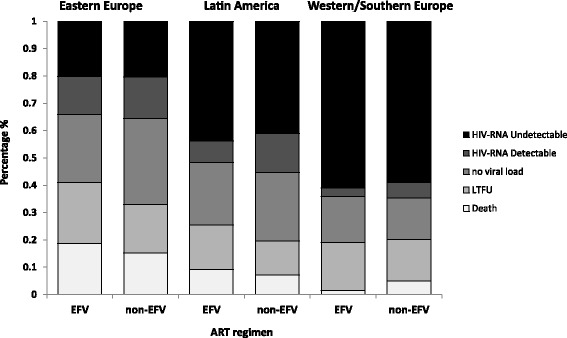
Outcomes at 12 months by region and ART regimen. EFV: Efavirenz, LTFU: loss to follow-up. HIV-RNA undetectable: VL < 400 copies/ml at 12 months

Mortality, the proportion of patients with undetectable viral loads, and loss to follow up was similar for patients receiving efavirenz and non-efavirenz ART regimens, irrespective of whether patients were ART-naïve or ART-experienced at TB diagnosis (Table 2). Clinical outcomes differed significantly across regions, although within regions, similar outcomes were observed for patients receiving efavirenz and non-efavirenz ART regimens (Fig. 1). The highest proportion of deaths and the lowest proportion of patients with undetectable HIV viral loads at 12 months were observed in EE. Many patients in all regions had no viral load information in the time window 8–12 months following TB diagnosis: 25% in EE, 17% in WE, and 23% in LA) for those receiving an efavirenz-containing regimen (*p* < 0.01), and 32%, 15% and 25% for those taking a non-efavirenz regimen, respectively (*p* < 0.01) (Fig. 1).

**Table 2:** Outcomes at 12 months in the entire cohort, naïve and non-naïve patients, by ART regimen

	All		ART naïve		ART non-naïve	
	Efavirenz (n = 647)	non Efavirenz (n = 318)	Efavirenz (n = 420)	non Efavirenz (n = 98)	Efavirenz (n = 227)	non Efavirenz (n = 220)
Death	84 (13%)	29 (9%)	51 (12%)	10 (10%)	33 (14%)	19 (9%)
LTFU	129 (20%)	50 (16%)	100 (24%)	20 (20%)	29 (13%)	30 (14%)
Undetectable HIV-RNA	220 (34%)	132 (41%)	134 (32%)	35 (36%)	86 (38%)	97 (44%)
Detectable HIV-RNA	67 (11%)	34 (11%)	44 (10%)	8 (8%)	23 (10%)	26 (12%)
Active no VL	147 (23%)	73 (23%)	91 (22%)	25 (25%)	56 (25%)	48 (22%)
*p* value	0.08		0.78		0.27	

Note: *p* values reflect the comparison of the overall distribution of outcomes for efavirenz- and non-efavirenz containing ART regimens

*LTFU* lost to follow-up, *HIV-RNA Undetectable* VL < 400 at 12 months, *VL* viral load

### Survival

By 12 months 113(12%) deaths had occurred: 84(13%) in the efavirenz group and 29(9%) in the non-efavirenz group. Using an inverse probability weighting method, no difference in survival (adjusted by region, age, gender, CD4 count at TB diagnosis, type of TB, route of HIV transmission, naïve status, rifamycin use at TB diagnosis, and MDR-TB) was observed between efavirenz and non-efavirenz treated individuals (Additional file 1: Figure S1). In adjusted Cox regression models stratified by ART status and use of rifamycin, the hazard ratio (HR) for death was 1.13 (95% CI: 0.72–1.78; *p* = 0.59) for efavirenz compared with non-efavirenz ART regimens (Table 3). In the same model, higher CD4 counts at TB diagnosis were associated with a lower risk of death (HR 0.27, 95%CI: 0.17–0.42, *p* < 0.001) for patients with CD4 of 350 cells/mm^3^ compared to those with CD4 50 cells/mm^3^, and naïve patients had a lower risk of death (HR 0.64, 95%CI: 0.43–0.96, *p* = 0.03) (Table 3). Gender, age, route of HIV transmission, disseminated TB, and MDR-TB were not significantly associated with mortality. Survival analysis stratified by ART status showed lower mortality among naïve compared with non-naïve patients, but there were no differences between patients receiving either efavirenz or non-efavirenz ART regimens (Additional file 1: Figure S2).

**Table 3:** Risk factors for death. Univariate and multivariate Cox models

	Univariate model		Multivariate model	
	Hazard Ratio (95% confidence interval)	*p*-value	Hazard Ratio (95% confidence interval)	p-value
Region		< 0.01		< 0.01
Eastern Europe	1		1	
Western/Southern Europe	0.16(0.08–0.31)		0.19 (0.09–0.39)	
Latin America	0.42(0.25–0.70)		0.44 (0.25–0.77)	
Age, years		0.69		0.87
30 vs 20	1.07(0.64–1.80)		1.12 (0.64–1.97)	
40 vs 20	1.07(0.47–2.42)		1.29 (0.53–3.13)	
50 vs 20	0.93(0.43–2.00)		1.53 (0.66–3.55)	
Male	1.26(0.81–1.95)	0.30	1.00 (0.62–1.60)	0.99
Disseminated TB	1.57(1.06–2.33)	0.03	1.38 (0.92–2.06)	0.12
IDU	1.89(1.31–2.74)	< 0.01	1.19 (0.78–1.83)	0.42
TB susceptibility		< 0.01		0.52
No MDR vs MDR	0.41(0.24–0.71)		0.72 (0.41–1.27)	
No Resistance test vs MDR	0.52(0.31–0.88)		0.84(0.49–1.46)	
Efavirenz	1.47(0.96–2.24)	0.07	1.13(0.72–1.78)	0.59
ART naïve	1.06(0.73–1.54)	0.74	0.64(0.43–0.96)	0.03
CD4^+^ at TB diagnosis (cells/mm^3^)		< 0.01		< 0.01
100 vs 50	0.63(0.53–0.76)		0.61(0.50–0.73)	
200 vs 50	0.35(0.25–0.51)		0.33(0.23–0.48)	
350 vs 50	0.27(0.17–0.42)		0.27(0.17–0.42)	

Note: Cox regression model in which patients were stratified by rifamicin use at TB diagnosis

Nine hundred sixty-five patients were included in this model. *IDU* Injection Drug User, *MDR-TB* Multi-drug resistant tuberculosis

### Marginal structural models

Of the 1389 patients in the prospective HIV:TB study 229(16.5%) never started ART and were therefore, only included in the marginal structural model. Of these 229 patients 92% were from EE, 4% from WE and 3% LA. Their median CD4 cell count was 149 cells/uL. 60% were IDU, 38% had MDR-TB and 65% had disseminated TB (see Additional file 1: Table S2). Overall 117(51%) died, and at 12 months after TB diagnosis 75(33%) were lost to follow up. Using the marginal structural model the odds of death (OR) for those in receipt of an efavirenz-containing ART regimen was 0.82 (95%CI: 0.66–1.02, *p* = 0.08, when compared to those receiving a non-efavirenz containing regimen. After truncating weights for different percentiles, such as 95%, the mortality odds remained non-significant (OR 0.85 (95%CI: 0.67–1.07, *p* = 0.18). These results, obtained using this model were consistent with the other statistical techniques used in this study. Furthermore exploration, in an analysis restricted to patients starting efavirenz compared with those starting a ritonavir-boosted protease inhibitor regimen only, showed that the odds of death for those who started an efavirenz-containing ART regimen was statistically better (OR 0.76, 95%CI: 0.60–0.95, *p* = 0.01).

### Sensitivity analysis for mortality among ART-naïve patients

This analysis included 518 ART-naïve patients: 420 (81%) receiving efavirenz-based ART and 98 (19%) non-efavirenz containing ART. Adjusted survival curves are shown in Additional file 1: Figure S3 and the Cox model results are shown in Additional file 1: Table S1. In the marginal structural model, odds of death were better in those receiving efavirenz-based ART: OR 0.55 (95%CI: 0.36–0.85, *p* < 0.01). Truncating at other percentiles showed similar results. Additional file 1: Figure S4 and Table S3 show the results for ART-non naïve patients.

### Virological suppression

Among 453 of the original 965 patients who still were alive and in care at 12 months, and who had viral load information available, 352 (77%) patients had undetectable plasma HIV viral loads (less than 400 copies/ml). The proportion of patients with virological suppression was less frequent in EE, regardless of the ART regimen, compared to the other regions. Distribution by ART regimen and treatment experience is shown in Table 2. In an adjusted Cox model, the hazard ratio (HR) for virological suppression was 0.97 (95%CI: 0.76–1.22: *p* = 0.77) for efavirenz, compared with non-efavirenz containing ART regimens. Gender, age, route of HIV transmission, disseminated TB, rifamycin use, being ART-naïve, and having documented MDR-TB were not significantly associated with virological suppression (Table 4).

**Table 4:** Factors associated with virological success

	Univariate model		Multivariate model	
	Hazard Ratio (95% confidence interval)	*p*-value	Hazard Ratio (95% confidence interval)	*p*-value
Region		0.07		< 0.01
Eastern Europe	1		1	
Western/Southern Europe	0.99(0.77–1.27)		1.03 (0.75–1.41)	
Latin America	1.29(0.97–1.73)		1.42 (1.04–1.94)	
Age, years		0.59		0.87
30 vs 20	1.13(084–1.52)		1.12 (0.83–1.51)	
40 vs 20	1.24(0.76–2.02)		1.22 (0.74–2.01)	
50 vs 20	1.26(0.80–2.00)		1.28 (0.80–2.06)	
Male	1.01(0.81–1.27)	0.91	0.93 (0.73–1.18)	0.53
Disseminated TB	0.90(0.73–1.12)	0.34	0.86 (0.69–1.07)	0.17
IDU	1.07(0.85–1.34)	0.58	1.16 (0.87–1.55)	0.30
TB susceptibility		0.79		0.84
No MDR vs MDR	1.07(0.65–1.75)		1.05(0.62–1.78)	
No Resistance test vs MDR	1.04(0.63–1.72)		0.98(0.58–1.66)	
Rifamicin	1.25(0.79–1.97)	0.33		
Efavirenz	1.05(0.84–1.30)	0.68	0.97(0.76–1.22)	0.77
ART naïve	1.11(0.90–1.37)	0.33	1.09(0.86–1.39)	0.44
CD4^+^ at TB diagnosis (cells/mm^3^)		0.48		0.41
100 vs 50	0.99(0.90–1.08)		0.99(0.90–1.10)	
200 vs 50	0.96(0.79–1.16)		0.98(0.79–1.20)	
350 vs 50	0.91(0.73–1.14)		0.92(0.73–1.17)	

Note: Only 453 patients in care at 12 months were included in this model. Patients without HIV-RNA information were excluded, *n* = 220

*IDU* Injection Drug User, *MDR-TB* Multi-drug resistant tuberculosis

### Loss to follow-up

Of the 852 patients included in this analysis, which only excluded patients who died, 179 (21%) were lost to follow up at 12 months (Table 2). In a Cox model, adjusted for gender, age, region, CD4 count at diagnosis of TB, loss to follow-up was similar among those receiving an efavirenz-containing regimen of ART, when compared with non-efavirenz containing regimens: HR 1.17 (95% CI: 0.81–1.67: *p* = 0.40). However, a higher risk of loss to follow-up was found in patients from EE, compared to patients from other regions (Table 5).

**Table 5:** Risk factors for loss to follow-up

	Univariate model		Multivariate model	
	Hazard Ratio (95% confidence interval)	p-value	Hazard Ratio (95% confidence interval)	*p*-value
Region		< 0.01		< 0.01
Eastern Europe	1		1	
Western/Southern Europe	0.59(0.41–0.84)		0.77(0.50–1.16)	
Latin America	0.58(0.39–0.87)		0.64(0.41–1.00)	
Age, years		0.59	a	
30 vs 20	1.20(0.79–1.83)			
40 vs 20	1.20(0.63–2.29)			
50 vs 20	0.82(0.85–1.50)			
Male	1.00(0.73–1.40)	0.96	0.90(0.64–1.28)	0.56
Disseminated TB	0.89(0.66–1.19)	0.44	0.93(0.68–1.26)	0.62
IDU	1.25(0.93–1.69)	0.13	0.97(0.68–1.37)	0.85
TB susceptibility		0.52		0.84
No MDR vs MDR	0.72(0.41–1.26)		0.92(0.51–1.65)	
No Resistance test vs MDR	1.19(0.69–2.05)		1.45(0.83–2.52)	
Rifamicin	0.84(0.50–1.41)	0.51	a	
Efavirenz	1.38(0.99–1.91)	0.06	1.17(0.81–1.67)	0.40
ART naïve	1.94(1.42–2.65)	< 0.01	a	
CD4^+^ at TB diagnosis (cells/mm^3^)		0.48		0.41
100 vs 50	0.97(0.86–1.10)		0.99(0.90–1.10)	
200 vs 50	0.95(0.73–1.25)		0.98(0.79–1.20)	
350 vs 50	0.98(0.72–1.34)		0.92(0.73–1.17)	

Note: Multivariate Cox model regression stratified by ryfamicin use, naïve condition and age. ^a^For these variables the model was stratified so we do not have specific hazard ratios. Eight hundred fifty-two patients included, only people who died at 12 months were excluded in this model

## Discussion

In this prospective observational study, there were major differences in outcomes that were predicated by patients’ region of residence. The mortality in Eastern Europe was significantly higher than in other regions, as described previously [3, 34, 35], but this did not differ significantly according to ART regimen (either efavirenz or non-efavirenz containing ART). Although ART-naïve patients had slightly better survival in comparison to ART non-naïve patients, this was similar for patients receiving efavirenz and non-efavirenz containing ART regimens. Furthermore, the proportions of patients who became lost to follow up and who had undetectable viral loads at 12 months following diagnosis of TB did not differ according to whether efavirenz was included in the ART regimen.

The observed similar survival in patients treated with efavirenz or non-efavirenz regimens may have several explanations, including that the non-efavirenz group was heterogeneous. Although inverse probability weighting was used to balance the differences in the co-variables between groups, it is possible that not all confounders including socio-demographic factors, comorbidities and/or concomitant (opportunistic) infections, diagnostic delay, and access to anti-TB drug susceptibility tests were included. In contrast with previously described findings in this cohort, MDR-TB was not associated with mortality at 12 months, probably because those with MDR-TB were less likely to start antiretroviral therapy and were therefore not included in the current study: moreover, a resistance test was performed in only 54% of the selected population. In the main model used, no differences in mortality between regimes was observed, but in an explorative marginal structural model restricted just to patients treated with a boosted protease inhibitor or efavirenz, survival was statistically different, favoring the group receiving efavirenz-based ART. This finding may reflect poorer tolerability of protease inhibitors, greater frequency of drug-drug interactions, or previous virological failure that could additionally impact on mortality. Surprisingly, there was a high proportion of subjects receiving PI-based ART as their first regimen (57%). This group of patients were mostly in the Eastern European region (61%), and most likely the reason for initiating ART with a PI-containing regimen was because of the local availability. Moreover, only 22% of those on non-efavirenz regimens received integrase inhibitors, a majority from Western Europe. Any inferences based on outcomes with this class of ART should be interpreted with caution as the study was not designed to directly compare integrase inhibitors with efavirenz, and additionally, the main analysis may have lacked power to detect a survival benefit.

The proportion of patients who were lost to follow up was high, especially among patients from Eastern Europe, irrespective of ART regimen. This is consistent with findings from other studies in countries with a high burden of TB [36]. Higher rates of loss to follow up in some other studies may be explained by differences in patient populations, health care provision models including more assertive outreach to patients who have dropped out of care. Although frequency of patients with virological suppression at 12 month was overall acceptable (77%), the proportion of plasma HIV-RNA determinations was heterogeneous between regions. Nevertheless, if the goal is to reach the 90–90-90 target, this rate of virological suppression is still some way from being accomplished. No clinical factors associated with an undetectable HIV viral load were found in this study, however it was less likely to be achieved in Eastern Europe. In general, unfavorable outcomes such as loss to follow-up, proportion of detectable viral load and mortality were more frequent in the Eastern European region, which is consistent with previous publications from our study [2, 3]. Factors such as characteristics of the HIV population (i.e. the high proportion of IDU), education and health care barriers, less access to HIV monitoring such as HIV-RNA determinations, and the feasibility of implementing current recommendations on ART initiation in the context of an AIDS-defining event [37], might have an important role when patients are compared with other regions with limited healthcare resources, such as Latin America.

Our study has several limitations. These include the relatively short follow-up period, differences between regions in terms of routine care such as access to HIV-RNA determinations, heterogeneity of use of ART, availability of rifabutin and integrase inhibitors, and timing of ART initiation at TB diagnosis. In terms of data availability, we recognize limitations in information regarding reasons for initiating or changing specific ART regimens, which did not allow us to accurately distinguish between changes due to adverse effects or changes due to ART failure and we cannot exclude the possibility that some naïve patients might have been receiving ART – although this was not detected during our quality assurance procedures. However, this is the largest multi-regional TB:HIV cohort and the first to comprehensively compare the concomitant use of TB treatment and efavirenz and non-efavirenz containing ART regimes. Using marginal structural models, there was a slightly better outcome for patients treated with efavirenz-containing ART, although this was not consistent in all analyses and therefore not categorical. These results warrant further analyses.

## Conclusions

In this multi-centre, multi-region cohort study, overall, poor outcomes were more frequent in Eastern Europe. Use of efavirenz or non-efavirenz containing ART regimens did not impact on mortality, virological suppression, or loss to follow-up at 12 months following diagnosis of TB. Considering these differences between regions irrespective of ART regimen, more, prospective studies are needed, particularly in resource-constrained regions in which access to rifabutin and integrase inhibitors is still limited.

## Additional file

Additional file 1: Supplementary material response to ART in HIV-TB. **Figure S1.** Probability of death in efavirenz and non-efavirenz containing ART groups by inverse probability weighting method. **Figure S2.** Cox adjusted survival by ART regimen during the first year stratified by naïve status. **Figure S3.** Adjusted probability of death in the efavirenz group compared to the non-efavirenz group for naïve patients only. **Table S1.** Risk factors for death in naïve patients. **Figure S4.** Adjusted probability of death in the efavirenz group compared to the non-efavirenz group for non-naïve patients only. **Table S2.** Demographic and clinical characteristics of patients who never started ART at the time of starting TB treatment. **Table S3.** Risk factors for death in non-naïve patients. (DOCX 1262 kb)
