# Supplementary material for: Differences in response to antiretroviral therapy in HIV-positive patients being treated for tuberculosis in Eastern Europe, Western Europe and Latin America
Source: BMC Infect Dis. 2018 Apr 23;18:191. doi: 10.1186/s12879-018-3077-x (PMC5914014; doi:10.1186/s12879-018-3077-x)
Supplement: Supplementary file 1 — Supplementary material response to ART in HIV-TB. Figure S1. Probability of death in efavirenz and non-efavirenz containing ART groups by inverse probability weighting method. Figure S2. Cox adjusted survival by ART regimen during the first year stratified by naïve status. Figure S3. Adjusted probability of death in the efavirenz group compared to the non-efavirenz group for naïve patients only. Table S1. Risk factors for death in naïve patients. Figure S4. Adjusted probability of death in the efavirenz group compared to the non-efavirenz group for non-naïve patients only. Table S2. Demographic and clinical characteristics of patients who never started ART at the time of starting TB treatment. Table S3. Risk factors for death in non-naïve patients. (DOCX 1262 kb) [file 12879_2018_3077_MOESM1_ESM.docx]

**Additional file 1**

**Figure S1. Probability of death in efavirenz and non-efavirenz containing ART groups by inverse probability weighting method.
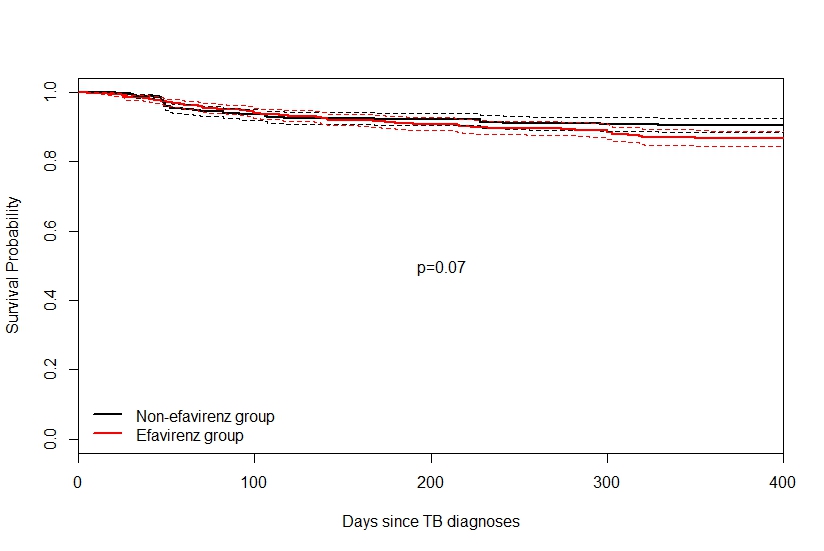
**

*Note:* Adjusted by gender, age, region, MDR-TB, rifamycin use, IDU status, and naïve condition and TB type (disseminated vs not disseminated). Dotted lines are the confidence interval for the survival estimated in each ART group*.* IDU: Injecting Drug Use, MDR-TB: Multi-drug resistant tuberculosis (n=965).

There is no significant difference in survival between EFV and non-EFV groups (p=0.07).

**Figure S2. Cox adjusted survival by ART regimen during the first year stratified by naïve status.**

**
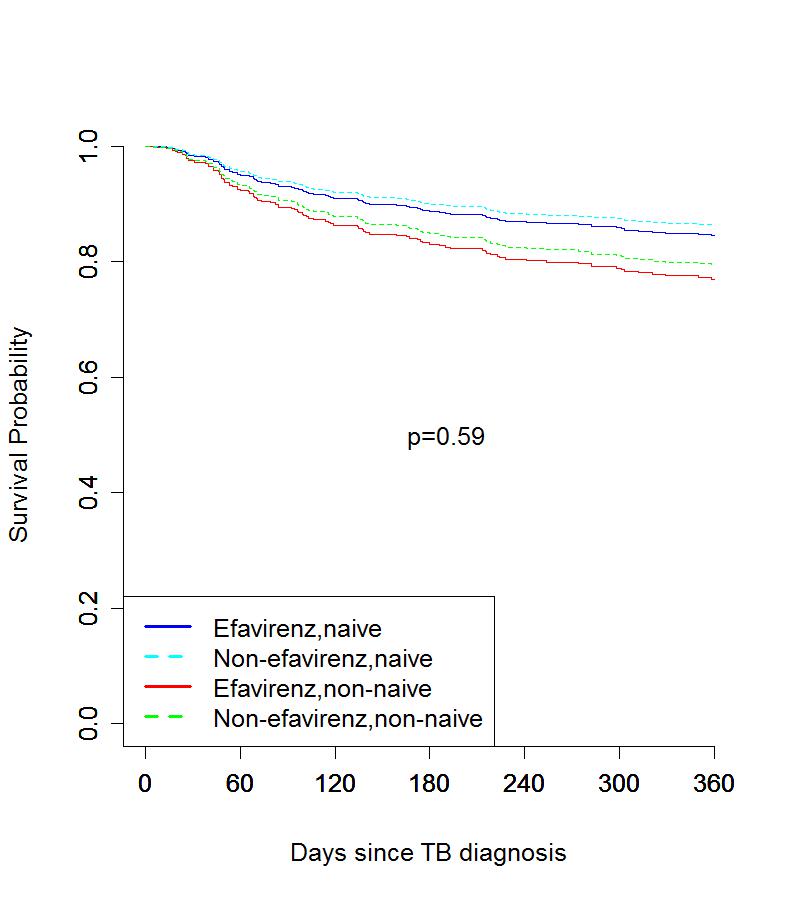
**

*Note:* Co-variables fixed at: 37 years, Eastern Europe, male, CD4 100 cells/mm^3^ at TB diagnosis, IDU, MDR, Disseminated TB and rifamycin use at TB diagnosis.

There is no significant difference between EFV and non-EFV groups (p=0.59), but there is a difference between naïve status and non-naïve status (p=0.03).

**Figure S3. Adjusted probability of death in the efavirenz group compared to the non-efavirenz group for naïve patients only.**

**
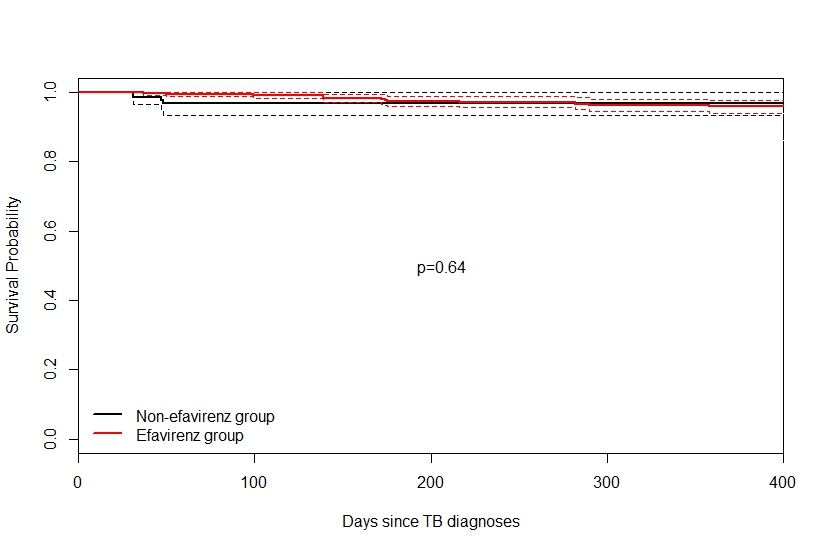
**

*Note:* Adjusted by gender, age, region, MDR-TB, rifamycin use, IDU status and TB type (disseminated vs non-disseminated). Dotted lines are the 95% confidence intervals for survival curves estimated in each ART group.

There is no significant difference in mortality between EFV and non-EFV groups comparing naïve patients only (p=0.64).

**Table S1. Risk factors for death in naïve patients.**

|  | Univariate  model |  | Multivariate  model |  |
| --- | --- | --- | --- | --- |
|  | Hazard Ratio  (95% confidence interval) | p-value | Hazard Ratio  (95% confidence interval) | p-value |
| Region |  | <0.01 |  | 0.07 |
| Eastern Europe | 1 |  | 1 |  |
| Western Europe | 0.08(0.02-0.35) |  | 0.10 (0.02 -0.45) |  |
| Latin America | 0.39(0.18-0.87) |  | 0.46 (0.19-1.08) |  |
| Age, years |  | 0.25 |  | 0.67 |
| 30 vs 20 | 1.35(0.61-3.00) |  | 1.42 (0.58 -3.45) |  |
| 40 vs 20 | 1.42(0.40-5.05) |  | 1.68 (0.40-7.02) |  |
| 50 vs 20 | 0.88(0.27-2.82) |  | 1.37 (0.37-5.10) |  |
| Male | 1.00(0.53-1.88) | 0.98 | 0.75 (0.39-1.46) | 0.39 |
| Disseminated TB | 1.93(1.09-3.42) | 0.02 | 2.08 (1.15-3.78) | 0.01 |
| IDU | 2.23(1.34-3.73) | <0.01 | 1.34 (0.75-2.42) | 0.32 |
| TB susceptibility |  | 0.08 |  | 0.40 |
| No MDR vs MDR | 0.45(0.22-0.92) |  | 0.61 (0.29-1.28) |  |
| No Resistance test vs MDR | 0.54(0.27-1.07) |  | 0.65(0.32-1.34) |  |
| Rifamycin | 0.51(0.23-1.12) | 0.09 | 0.58(0.25-1.31) | 0.18 |
| Efavirenz | 1.17(0.59-2.30) | 0.64 | 1.13(0.55-2.33) | 0.73 |
| CD4^+^ at TB diagnosis  (cells/mm^3^) |  | 0.01 |  | 0.04 |
| 100 vs 50 | 0.78(0.59-1.02) |  | 0.79(0.59-1.03) |  |
| 200 vs 50 | 0.48(0.30-0.79) |  | 0.52(0.32-0.86) |  |
| 350 vs 50 | 0.25(0.07-0.85) |  | 0.29(0.08-1.05) |  |

*Note:* Cox model regression stratified by ART regimen. 518 patients included*.* IDU: Injection Drug User. MDR-TB: Multi-drug resistant tuberculosis.

**Figure S4. Adjusted probability of death in the efavirenz group compared to the non-efavirenz group for non-naïve patients only.
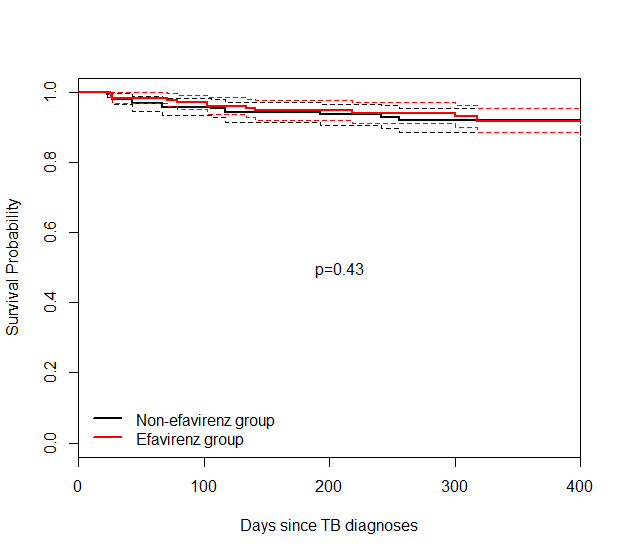
**

*Note:* Adjusted by gender, age, region, MDR-TB, rifamycin use, IDU status and TB type (disseminated vs non-disseminated). Dotted lines are the 95% confidence intervals for survival curves estimated in each ART group.

There is no significant difference in survival between EFV and non-EFV groups comparing non-naïve patients only (p=0.43).

**Table S2. Demographic and clinical characteristics of patients who never started ART at the time of starting TB treatment.**

|  | Never on ARV  n=229 |
| --- | --- |
| Patient age at TB diagnosis (years)* | 34 (30 -40) |
| Male, n(%) | 162 (70%) |
| Ethnic group, n(%) |  |
| White | 192 (89%) |
| Hispanic | 6 (3%) |
| Black | 2 (1%) |
| Other | 16 (7%) |
| HIV risk, n(%) |  |
| IDU | 138 (60%) |
| non IDU | 91 (39%)) |
| Region, n(%)* |  |
| Eastern Europe | 211 (92.1%) |
| Western Europe | 10 (4.4%) |
| Latin America | 8 (3.5%) |
| CD4^+^ count (cells/mm^3^)* | 149 (46-367) |
| Missing CD4 counts, n (%) | 31 (13%) |
| HIV RNA (copies/mL), n(%) <400 | 3 (13%) |
| 400-10 0000 | 15 (6.5%) |
| >10 0000 | 99(43%) |
| Unknown | 112 (49%) |
| Haemoglobin (g/dL)* | 11.2 (9.2-13) |
| Disseminated TB, n(%) | 150 (65%) |
| Resistance test performed | 220 (96%) |
| MDR-TB, n(%)* | 84 (38%) |

*Statistically significant compared to patients on ART (p<0.001). Patients never on ART were younger than patients on ART, a higher proportion were from Eastern Europe, with higher CD4 counts, higher haemoglobin levels and lower MDR-TB rates

**Table S3. Risk factors for death in non-naïve patients.**

|  | Univariate  model |  | Multivariate  model |  |
| --- | --- | --- | --- | --- |
|  | Hazard Ratio  (95% confidence interval) | p-value | Hazard Ratio  (95% confidence interval) | p-value |
| Region |  | <0.01 |  | <0.01 |
| Eastern Europe | 1 |  | 1 |  |
| Western Europe | 0.19(0.08-0.45) |  | 0.19 (0.07 -0.49) |  |
| Latin America | 0.42(0.21-0.83) |  | 0.35 (0.16-0.75) |  |
| Age, years |  | 0.66 |  | 0.75 |
| 30 vs 20 | 0.90(0.46-1.79) |  | 1.13 (0.53 -2.39) |  |
| 40 vs 20 | 0.88(0.30-2.56) |  | 1.46 (0.44-4.79) |  |
| 50 vs 20 | 0.97(0.35-2.69) |  | 2.38 (0.76-7.50) |  |
| Male | 1.36(0.74-2.51) | 0.18 | 1.48 (0.76-2.88) | 0.25 |
| Disseminated TB | 1.26(0.73-2.18) | 0.42 | 0.83 (0.46-1.49) | 0.53 |
| IDU | 1.57(0.91-2.71) | 0.10 | 0.90 (0.46-1.76) | 0.76 |
| TB susceptibility |  | 0.09 |  | 0.56 |
| No MDR vs MDR | 0.36(0.15-0.86) |  | 0.59 (0.29-1.13) |  |
| No Resistance test vs MDR | 0.49(0.21-1.13) |  | 1.28(0.69-2.38) |  |
| Rifamycin | 0.42(0.22-0.79) | <0.01 | 1.31(0.52-3.26) | 0.11 |
| Efavirenz | 1.73(0.98-3.04) | 0.06 | 1.19(0.65-2.19) | 0.43 |
| CD4^+^ at TB diagnosis  (cells/mm^3^) |  | <0.01 |  | <0.01 |
| 100 vs 50 | 0.55(0.44-0.70) |  | 0.51(0.39-0.65) |  |
| 200 vs 50 | 0.23(0.13-0.40) |  | 0.19(0.10-0.34) |  |
| 350 vs 50 | 0.15(0.07-0.29) |  | 0.12(0.06-0.26) |  |

.
